# Recurrent urinary retention due to seminal vesicle cyst a report of rare entity

**DOI:** 10.1016/j.eucr.2023.102556

**Published:** 2023-09-09

**Authors:** Yazan Al-Dali, Ibrahim A. Khalil, Majd Alkabbani, Mohamed Hatem, Abdulhamed Mansour, Khalid Al Rumaihi

**Affiliations:** a College of Medicine, Qatar University, Doha, Qatar; b Department of Urology, Hamad Medical Corporation, Doha, Qatar

**Keywords:** Male pelvic cysts, Acute urinary retention, TRUS aspiration, Robotic pelvic cyst, Seminal vesical cyst

## Abstract

Male pelvic cyst rarely causes symptoms; here, we are presenting a case of a 48-year-old gentleman who presented with acute urinary retention stemming from a pelvic cyst. This presentation has been recurrent despite undergoing repeated TRUS aspiration of the cyst to relieve the symptoms. We performed a robotic pelvic cyst excision with peritoneal window in an attempt to cure the patient. Based on the MRI and histopathology, it was likely a seminal vesicle cyst that is causing these recurrent episodes. On 3 months follow-up, the patient was symptom free without any complaints.

## 1 Introduction

Male pelvic cystic masses are believed to be a rare condition.^1^ The prevalence is considered to be between 1 and 5% in the literature.^2^ They are generally classified according to their embryological origin (Müllerian ducts, prostatic utricle, ejaculatory ducts, seminal vesicle) and position related to the prostate gland (paramedian, median, lateral).^3^ This condition is usually asymptomatic and diagnosed incidentally on pelvic ultrasound.^3^ Symptoms that may arise from the cyst include recurrent urinary tract infection (UTI), infertility and lower urinary tract symptoms (LUTS). However, urinary retention is a rare presentation. In this case report, we present a case of a pelvic cyst in a 48-year-old gentleman that presented with recurrent acute urinary retention, including the clinical presentation, diagnostic evaluation, treatment, and outcome.

## 2 Case presentation

**Fig. 1:**
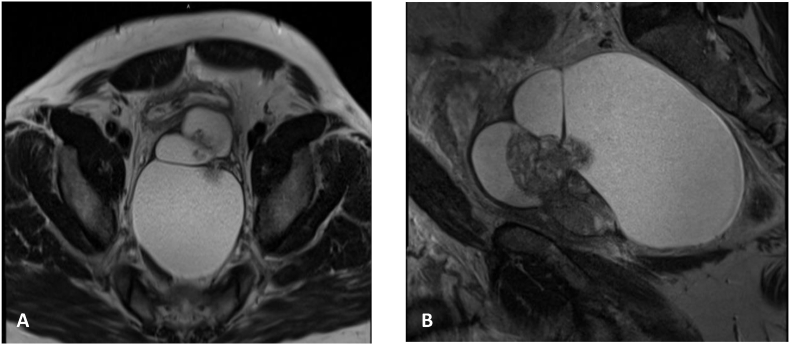
Initial MRI showing large seminal vesicle cyst on axial (A) and sagittal (B) sections.

**Fig. 2:**
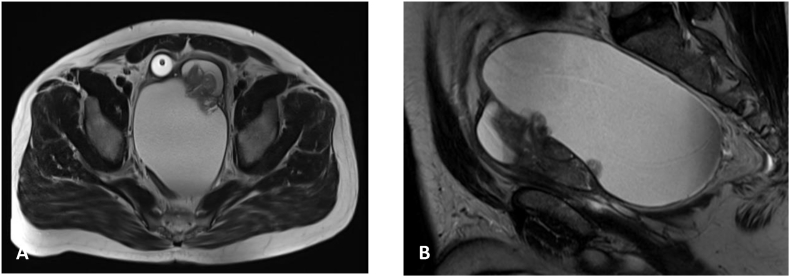
Follow up MRI showed large seminal vesicle cyst on axial (A) and sagittal (B) sections.

A 48-year-old gentleman with no comorbidities, presented to our emergency department (ED) with acute urinary retention for one day, a urethral catheter inserted. He complained of progressive obstructive lower urinary tract symptoms of weak stream, dysuria, and suprapubic pain. The patient did have a history of transrectal ultrasound guided (TRUS) aspiration of a seminal vesicle cyst before one year. The physical examination was unremarkable apart from digital rectal examination that showed large soft mass compressing the rectum, which hindered the assessment of the prostate. A magnetic resonance imaging (MRI) was performed which showed a pelvic cystic lesion, displacing the urinary bladder and prostate anteriorly and the rectum posteriorly, measuring 13× 9 x 9.5 cm, along with solid component that appears contiguous with enlarged transition zone of the prostate. The right and left seminal vesicles were identified along lateral margin of the cystic lesion on each side Fig. 1. Patient underwent redo TRUS guided aspiration of the cyst, which derived approximately 500mL of serous frothy fluid, Cytology yielded red blood cells (RBCs), mesothelial cells, and mixed inflammatory cells without sperms. The patient's symptoms improved and was able to pass urine smoothly post-procedure. Upon follow up, the patient developed another episode of acute urinary retention 3 months later, for which urethral catheter was inserted; MRI showed recurrence of the cyst Fig. 2. Due to failure of TRUS aspiration of the cyst with recurrent collection that caused obstruction, robotic pelvic cyst excision with peritoneal window was performed. The histopathology of the excised cystic mass showed fibrovascular tissue partly lined by mesothelial cells with chronic inflammation and fibrosis, along with tissue from seminal vesicle. Follow up 3 months after surgery, patient reported resolution of his symptoms.

## 3 Discussion

Male pelvic cysts (MPC) are considered to be a rare entity, with a prevalence of 1–5%.^2^ They can be classified as extraprostatic and intraprostatic.^2^ Extraprostatic cysts can be further classified based on their origin as seminal vesicle cysts, vas deferens cysts, or Cowper's duct's cysts, while the intraprostatic cysts can be classified according to their position within the prostate, as median, paramedian and lateral to the prostate. Mullerian duct and utricle cysts are median, ejaculatory duct cyst and urethral diverticulum are paramedian, prostatic retention cysts and cystic degeneration of benign prostatic hyperplasia (BPH) are lateral.^2^ In our case, the patient likely had a seminal vesicle as found in MRI and supported by histopathology reports.

Symptoms of MPC vary from asymptomatic to irritative or obstructive LUTS, infertility, chronic pelvic pain syndrome, painful ejaculation, or hematospermia.^4^ Infravesical obstruction causing acute urinary retention is an unusual presentation that indicates a huge cyst causing severe obstruction. To the best of our knowledge, this is the first case of a recurrent pelvic cyst presenting with recurrent episodes of acute urinary retention despite treatment.

Diagnosis of MPC is usually incidental.^2^ where a pelvic ultrasound is sufficient for the diagnosis. However, MRI is indicated for accurate localization and identifying the origin of the cyst. The possibility of cancer should be excluded by imaging and histopathology.

The treatment of MPC revolves around decompression of the cyst. This could be done by TRUS guided aspiration, transurethral deroofing, and open or laparoscopic surgeries for cyst excision with risk of recurrence.^5^ Recurrence of the cyst after TRUS guided aspiration in our case made us opt for robotic pelvic cyst excision and peritoneal window to facilitate the drainage of the fluid.

## 4 Conclusion

Although rare, large pelvic cysts causing recurrent obstruction can result in a significant burden on patient's quality of life and health. The choice of treatment modality depends on the presentation, location, and size of the cyst, in a step wise approach starting from TRUS aspiration up to robotic excision.

## Declaration of competing interest

The authors declare that they have no financial or non-financial conflicts of interest related to the subject matter or materials discussed in the manuscript.

## Abbreviations

UTI: Urinary Tract Infection
LUTS: Lower Urinary Tract Symptoms
ED: Emergency Department
TRUS: Trans-Rectal Ultra-Sound
MRI: Magnetic Resonance Imaging
RBCs: Red Blood Cells
MPC: Male Pelvic Cyst
